# The Paris System for reporting urinary cytology improves the negative predictive value of high-grade urothelial carcinoma

**DOI:** 10.1186/s12894-022-01005-8

**Published:** 2022-04-05

**Authors:** Mari Yamasaki, Rikiya Taoka, Kazuya Katakura, Toru Matsunaga, Naoya Kani, Tomoko Honda, Satoshi Harada, Yoichiro Tohi, Yuki Matsuoka, Takuma Kato, Homare Okazoe, Hiroyuki Tsunemori, Nobufumi Ueda, Reiji Haba, Mikio Sugimoto

**Affiliations:** 1 Department of Urology, Faculty of Medicine, Kagawa University, Japan, 1750-1, Ikenobe, Miki-cho, Kita-gun, Kagawa Japan; 2 Department of Diagnostic Pathology, University Hospital, Faculty of Medicine, Kagawa University, Japan, 1750-1, Ikenobe, Miki-cho, Kita-gun, Kagawa Japan

**Keywords:** Urothelial carcinoma, High-grade urothelial carcinoma, Urine cytology, The Paris System for reporting urinary cytology, Negative predictive value

## Abstract

**Background:**

The Paris System (TPS) for reporting urinary cytology differs from conventional systems (CS) in that it focuses on the diagnosis of high-grade urothelial carcinoma (HGUC). This study investigated the impact of TPS implementation on the diagnostic accuracy of HGUC by comparing it with our institutional CS.

**Methods:**

A total of 649 patients who underwent transurethral resection of bladder tumor (TURBT) between January 2009 and December 2020 were included in this study. Our institution adopted TPS to report urinary cytology in February 2020. The diagnostic accuracy of HGUC in preoperative urinary cytology was compared with the presence or absence of HGUC in resected specimens of TURBT before and after TPS implementation.

**Results:**

After implementing TPS in urinary cytology, 89 patients were reviewed and compared with 560 patients whose urinary cytology was diagnosed by CS. TPS and CS for detecting HGUC had 56.0% and 58.2% sensitivity, 97.8% and 91.2% specificity, and 93.3% and 87.9% positive predictive values, respectively. There were no significant differences between TPS and CS in terms of sensitivity, specificity, and positive predictive value for HGUC (*P* = 0.83, 0.21, 1.00). On the other hand, the negative predictive value for HGUC using TPS was 80.0%, which was significantly higher than that of CS (66.4%, *P* = 0.04) The multivariate logistic regression analysis indicated that not using TPS was one of the independent predictive factors associated with false-negative results for HGUC (odds ratio, 2.26; 95% confidence interval, 1.08–4.77; *P* = 0.03).

**Conclusion:**

In instances where urinary cytology is reported as negative for HGUC by TPS, there is a low probability of HGUC, indicating that TPS has a potential diagnostic benefit.

## Background

Based on the latest Global Cancer Incidence, Mortality And Prevalence data, bladder cancer (BC) is the 10th most common form of cancer worldwide, with an estimated 573,000 new cases in 2020 [1]. Approximately 75% of newly diagnosed BC cases are non-muscle-invasive BC (NMIBC) [2]. In clinical practice, NMIBC is treated with transurethral resection of bladder tumor (TURBT) followed by intravesical therapy, depending on the risk of recurrence and progression [3]. However, NMIBC recurs in approximately 50% of the cases [2]. Therefore, patients with NMIBC need surveillance using regular cystoscopy and urinary cytology for at least 5 years after the initial treatment [3].

Some histological types of BC contain pathologically different properties with varying clinical courses. In 2004, the World Health Organization/ International Society of Urological Pathology consensus classification system for papillary urothelial neoplasms of the urinary bladder was published [4]. The system classifies urothelial cancer (UC) into two main types: low-grade UC (LGUC) and high-grade UC (HGUC) [5]. Clinically, although LGUC has a low malignant potential, HGUC has a risk of disease progression and metastases, followed by death [6, 7]. Therefore, HGUC cannot be ignored in patients with BC.

Urinary cytology is a convenient screening tool for UC [3, 8]. However, the terminology for reporting urinary cytology has not been standardized. The Paris System (TPS) for reporting urinary cytology is a recently established international system for diagnosing urinary tract cytology specimens [9]. TPS differs from conventional systems (CS) in that it focuses on the diagnosis of HGUC. Effective detection of HGUC, which has the potential to progress and metastasize, is crucial for patients with suspected HGUC of the bladder. Therefore, TPS, which focuses on the diagnosis of HGUC, may benefit patients more than CS [10, 11]. However, there are few reports on the use of TPS for HGUC detection. This study, therefore, aimed to reveal the impact of TPS implementation for urinary cytology diagnosis on the diagnostic accuracy of HGUC in real clinical situations by comparing it with CS.

## Methods

### Study population and design

From January 2009 to December 2020, 755 patients underwent TURBT at Kagawa University Hospital. Of these, 21 patients who were pathologically diagnosed with non-urothelial malignancies and 85 patients whose urinary cytology had not been evaluated before TURBT were excluded. A total of 649 patients were included in the study. Our institution adopted TPS to report urinary cytology in February 2020. Before that, we used CS based on the Papanicolaou-stained urinary cytology system. Therefore, 560 patients who underwent urinary cytology before February 2020 were diagnosed with CS, while the subsequent 89 patients used TPS.

Urine specimens were the voided urine samples on the day before TURBT. The CS had five classes: class 1, inadequate or absence of suspicious cells; class 2, atypical cells but not malignant; class 3, cells suspected of being malignant but not confidently; class 4, suspected malignant cells; and class 5, malignant cells. We defined the criteria for positive urinary cytology as class 4 or 5, and negative urinary cytology was classified as class 1 or 2 in the CS. On the other hand, TPS required four categories for reporting urinary cytology: negative for HGUC (NHGUC), atypical urothelial cell, suspicious for HGUC (SHGUC), and HGUC. This study defined SHGUC and HGUC as positive urinary cytology and NHGUC as negative urinary cytology in TPS. We retrospectively compared the positive or negative results of preoperative urinary cytology in the presence or absence of HGUC in resected specimens of TURBT.

### Data collection

In addition to reports of histopathological diagnosis of the resected specimen and preoperative urinary cytology, age, sex, prior recurrence status, tumor appearance, tumor number, largest tumor diameter, tumor grade, pathological T-stage, and presence of carcinoma in situ (CIS) were retrospectively investigated. This study was conducted according to the principles outlined in the Declaration of Helsinki (as revised in Fortaleza, Brazil, October 2013), and these surveys were performed with the approval of the Ethics Committee of Kagawa University (permission number: 2020-070). The need for informed consent was waived by the Ethics Committee, Faculty of Medicine, Kagawa University because of the retrospective nature of this study.

### Statistical analysis

The diagnostic accuracy of HGUC was calculated as the sensitivity, specificity, negative predictive value (NPV), and positive predictive value (PPV). The Mann–Whitney U test or Fisher’s exact test was used to compare the clinical characteristics and diagnostic accuracy index between TPS and CS groups. Multivariate analysis with a logistic regression model was performed to determine an independent predictive factor for NPV errors. The number of variables incorporated into the multivariate analysis was determined based on the number of events. All statistical analyses were performed using SPSS for Windows version 12 (SPSS Inc., Chicago, IL, USA). Statistical significance was set at *P* < 0.05.

## Results

### Patient characteristics

Data from a total of 89 patients diagnosed with BC after implementing TPS were reviewed and compared with 560 patients whose urinary cytology was diagnosed by CS. Table 1 shows the patients’ characteristics in TPS and CS groups. The proportion of recurrent BC in the TPS group was lower than that in the CS group (23.6% vs. 39.5%, *P* < 0.01). The proportion of TURBT history including the second TURBT within 90 days was higher in the TPS group than in the CS group (30.3% vs. 14.6%, *P* < 0.01). In addition, the TPS group had a larger tumor size than the CS group (*P* = 0.05). By contrast, there were no significant differences in age, sex, tumor appearance, tumor number, presence of high-grade tumor, pathological tumor stage, and presence of CIS between the two groups.

**Table 1:** Patient characteristics

Variables		CS (n = 560)	TPS (n = 89)	*P* values*
Mean age	Years (range)	73.1 (31–100)	73.5 (26–93)	0.39
Sex				
Male	No. (%)	487 (87.0)	77 (86.5)	0.91
Female	No. (%)	73 (13.0)	12 (13.5)	
Prior recurrence status				
Primary	No. (%)	339 (60.5)	68 (76.4)	< 0.01
Recurrent	No. (%)	221 (39.5)	21 (23.6)	
Prior TUR within 90 days				
Yes	No. (%)	82 (14.6)	27 (30.3)	< 0.01
No	No. (%)	478 (85.4)	62 (69.7)	
Tumor appearance				
Papillary	No. (%)	478 (85.4)	78 (87.6)	0.57
Solid	No. (%)	82 (14.6)	11 (12.4)	
Tumor number				
Solitary	No. (%)	389 (69.5)	58 (65.1)	0.42
Multiple	No. (%)	171 (30.5)	31 (34.9)	
Largest tumor diameter				
< 3 cm	No. (%)	532 (95.0)	80 (89.9)	0.05
≥ 3 cm	No. (%)	28 (5.0)	9 (10.1)	
High-grade tumor				
Yes	No. (%)	313 (55.9)	42 (47.2)	0.13
No	No. (%)	247 (44.1)	47 (52.8)	
Pathological tumor stage (Benign vs pT+)				
Benign	No. (%)	108 (19.2)	25 (28.1)	0.06
pTis	No. (%)	49 (8.8)	8 (9.0)	
pTa	No. (%)	243 (43.4)	31 (34.8)	
pT1	No. (%)	117 (20.9)	16 (18.0)	
≥ pT2	No. (%)	43 (7.7)	9 (10.1)	
Concomitant CIS				
Yes	No. (%)	120 (21.4)	18 (20.2)	0.79
No	No. (%)	440 (78.6)	71 (79.8)	

*CS* conventional systems, *TPS* The Paris System, *TUR* transurethral resection, *CIS* carcinoma in situ

*P values were estimated using using the Mann–Whitney U test or the Fisher’s exact test

### Urinary cytology and histopathological diagnosis

Table 2 shows a summary of the urinary cytology and histopathological diagnoses for each TPS and CS group. The proportion of patients who were diagnosed with SHGUC and HGUC in the TPS group was 16.9%, which was significantly lower than that of patients with positive urinary cytology in the CS group (26.6%, *P* = 0.04). Of 560 patients in the CS group, 313 (55.9%) were histologically diagnosed with HGUC in the resected specimens. Of those, 94 (30.0%) patients were diagnosed with negative urinary cytology and 131 (41.9%) patients were diagnosed with positive urinary cytology. In contrast, of 89 patients in the TPS group, 42 (47.2%) were histologically diagnosed with HGUC. Of these, 11 (26.2%) patients were diagnosed with negative urinary cytology and 14 (33.3%) patients were diagnosed with positive urinary cytology.

**Table 2:** Summary of cytological and histopathological diagnoses

Variables	Histopathological diagnosis			
	Negative	LGUC	HGUC	Total
CS				
Class 1	16	31	11	58 (10.4)
Class 2	63	76	83	222 (39.6)
Class 3	19	24	88	131 (23.4)
Class 4	2	3	32	37 (6.6)
Class 5	8	5	99	112 (20.0)
Total	108 (19.3)	139 (24.8)	313 (55.9)	560 (100.0)
TPS				
NHGUC	23	21	11	55 (61.8)
AUC	1	1	17	19 (21.3)
SHGUC	0	0	3	3 (3.4)
HGUC	1	0	11	12 (13.5)
Total	25 (28.1)	22 (24.7)	42 (47.2)	89 (100.0)

Data are shown as no. or no (%)

*CS* conventional systems, *TPS* The Paris system, *LGUC* low-grade urothelial carcinoma, *HGUC* high-grade urothelial carcinoma, *NHGUC* negative for high-grade urothelial carcinoma, *AUC* atypical urothelial cells, *SHGUC* suspicious for high-grade urothelial carcinoma, *HGUC* high-grade urothelial carcinoma

### Sensitivity, specificity, PPV, and NPV of urine cytology for HGUC

Table 3 summarizes the detection of HGUC in TPS and CS groups. There were no significant differences between TPS and CS groups in terms of sensitivity, specificity, and PPV for HGUC. Contrastingly, the TPS group had a significantly higher NPV for HGUC than the CS group (80.0% vs. 66.4%, *P* = 0.04). A total of 105 patients had false-negative urinary cytology results for HGUC.

**Table 3:** The detection ability of high-grade urothelial carcinoma

	CS	TPS	*P* values*
Sensitivity (%)	58.2	56.0	0.83
Specificity (%)	91.2	97.8	0.21
PPV (%)	87.9	93.3	1.00
NPV (%)	66.4	80.0	0.04

*CS & TPS* Conventional & The Paris systems for reporting urinary cytology, *PPV & NPV* positive & negative predictive value

*P value was estimated using the Fisher’s exact test

Multivariate logistic regression analysis, which included prior recurrence status, prior TURBT within 90 days, and intraoperative factors such as tumor appearance, tumor number, and largest tumor diameter, revealed that the evaluation using CS rather than that using TPS was an independent predictive factor associated with false-negative results for HGUC (odds ratio, 2.26; 95% confidence interval, 1.08–4.77; *P* = 0.03; Table 4).

**Table 4:** Multivariate analysis for the prediction of false-negatives for high-grade urothelial carcinoma

Variables	Multivariate		
	ORs*	95% CI	*P* values*
Evaluated using CS rather than TPS	2.26	1.08–4.77	0.03
Prior recurrence status	0.47	0.27–0.79	< 0.01
Prior TUR within 90 days	0.71	0.36–1.38	0.31
Multiple tumor	1.59	0.91–2.78	0.11
Solid tumor	1.99	1.03–5.00	< 0.01
Large tumor (≥ 3 cm)	2.27	0.49–8.07	0.33

*CI* confidence, *OR* odds ratio, *CS* conventional systems, *TPS* The Paris system, *TUR* transurethral resection

*OR and *P* value were estimated using multivariate logistic regression analysis

## Discussion

TPS was created as an international form to standardize the reporting of urinary cytology [9]. TPS differs from CS in that it focuses on the diagnosis of HGUC. However, there are few reports on the ability of TPS to detect HGUC. This study compared TPS and CS for detecting HGUC in urine specimens before tumor resection and in resected tumor tissues. The results indicated that TPS was superior to CS in terms of its NPV for HGUC. HGUC is well known to be associated with a worse overall survival. Therefore, a high NPV for HGUC using TPS is clinically important for patients with suspected BC.

Urinary cytology is typically used to screen for UC in two clinical situations: when new-onset UC is suspected, such as in patients with unexplained hematuria, and during surveillance after BC treatment [12]. In particular, clinical guidelines suggest that cystoscopy is needed for at least 5 years for patients with a history of BC because half of the patients with BC will have an intravesical recurrence [2, 3]. However, cystoscopy is associated with physical pain and mental burden [13], which some patients experience during urinary tract infections [14]. If the high NPV of TPS can deny the presence of HGUC, and reduce additional examinations such as cystoscopy, it may improve patient quality of life and cost-effectiveness.

TPS defines standardized cytomorphologic and numerical criteria for its diagnostic categories, which stratify a patient’s risk for HGUC [9]. In other words, strict definitions of TPS may reduce the sensitivity of HGUC. In this study, 16.9% of patients were diagnosed with SHGUC and HGUC in the TPS group, which was significantly lower than that of patients with positive urinary cytology in the CS group. However, there was no difference in the sensitivity for HGUC between the TPS and CS groups. Recent reports demonstrated that the sensitivity for HGUC using TPS ranged from 83.3 to 87.1% [11, 15, 16], which were higher than that reported in this study. This study differs from other reports in that urine cytology was evaluated immediately before TURBT. Therefore, this study had a higher percentage of histologic HGUC, which may have led to a lower sensitivity of urine cytology for HGUC. On the other hand, the NPV was between 81.4% and 86.4% [11, 15, 16], which is similar to the value obtained in this study (80.0%). These data demonstrate that TPS can reduce the rate of unnecessary indeterminate diagnoses while maintaining excellent sensitivity for identifying HGUC.

As a screening test, it is imperative that urine cytology has a high NPV for HGUC. Our results indicated that TPS was superior to CS in terms of its NPV for HGUC. On the other hand, McIntire et al. compared the NPV of urinary cytology for detecting HGUC after TPS implementation to a pre-TPS cohort from the same institution and demonstrated that its NPV was unchanged after TPS implementation [17]. This is most likely due to their evaluation of all urine specimens rather than just urine specimens from patients undergoing TURBT. However, there is still debate as to whether TPS implementation can improve NPV for HGUC, and further study is needed.

Multivariable logistic regression analysis indicated that TPS may reduce false-negative results for HGUC. At the same time, recurrent tumors also reduced false-negatives for HGUC. Lee et al. analyzed the causes of false-negative cytology in HGUC cases [18]. The possible explanations were the overestimation of the grade by the pathologist and inflammation of the bladder. However, in this study, the risk of false-negative was low, even though inflammation due to prior transurethral surgery and subsequent intravesical instillation therapy may have affected the diagnosis of urinary cytology. On the other hand, the risk of false-negatives was found with solid tumors for HGUC as well as non-use of TPS. The relationship between solid tumors and false-negatives for HGUC may have been influenced by the smaller area of the tumor surface compared to papillary tumors. These results indicate the need for appropriate follow-up, including cystoscopy, for patients with a treatment history for solid tumors, instead of basing the results on a single negative urine cytology result. Thus, while a certain number of false negatives for HGUC are expected, serial urinary cytology may decrease false-negative results and improve sensitivity for detection [19]. In clinical practice, TPS should be used for urinary cytology because it yields fewer false-negative results than CS.

One of this study’s limitations is that cytological findings were interpreted by different pathologists. Recently, differences in the diagnosis of urinary cytopathology between pathologists have been reported [20]. Therefore, in this study, the accuracy of the diagnosis has been re-evaluated by another pathologist, and when the two diagnoses were different, a final diagnosis was made by discussion. However, further studies addressing interobserver variability should be conducted. In addition, urine collection methods, urine collection volumes, and urinary cell counts can affect the diagnostic quality of urinary cytology [9]. Since there was no information on urine specimens in this study, the effect of urine specimens on urinary cytology could not be investigated. The study’s retrospective nature was an additional limitation. However, to the best of our knowledge, this is the first study to consider various factors affecting the quality of urinary cytology, and demonstrate that the implementation of TPS in clinical practice may improve the NPV for HGUC.

## Conclusions

Our study revealed the impact of TPS implementation on the diagnostic accuracy of HGUC in real clinical situations by comparing it with CS. As a result, this study clearly indicates that, in instances where urinary cytology is reported as NHGUC by TPS, there is a low probability of HGUC. These results support the implementation of the TPS in clinical practice.

## Data Availability

The dataset generated and/or analyzed during the current study is not publicly available due to identifiable patient information but is available from the corresponding author upon reasonable request.

## Abbreviations

CIS: Carcinoma in situ
UC: Urothelial carcinoma
TPS: The Paris System
LGUC: Low-grade urothelial carcinoma
HGUC: High-grade urothelial carcinoma
CS: Conventional systems
NPV: Negative predictive value
PPV: Positive predictive value
TURBT: Transurethral resection of bladder tumor
SHGUC: Suspicious for high-grade urothelial carcinoma
NHGUC: Negative for high-grade urothelial carcinoma
BC: Bladder cancer
NMIBC: Non-muscle-invasive bladder cancer
